# Having the Heart to Exercise Control: Cardiac Interoception Influences Self‐Paced Exercise Regulation

**DOI:** 10.1002/ejsc.12263

**Published:** 2025-02-15

**Authors:** J. B. Butterworth, J. Dekerle, A. Greenhouse‐Tucknott, H. D. Critchley, N. J. Smeeton

**Affiliations:** ^1^ Fatigue and Exercise Laboratory School of Education, Sport and Health Sciences University of Brighton Brighton UK; ^2^ Cognitive Neuroimaging Unit (UNICOG) NeuroSpin, CEA Paris‐Saclay Gif‐sur‐Yvette Cedex France; ^3^ Department of Clinical Neuroscience Brighton and Sussex Medical School University of Sussex Brighton UK; ^4^ Neurobehavioural Clinic Sussex Partnership NHS Foundation Trust Brighton UK; ^5^ Sackler Centre for Consciousness Science University of Sussex Falmer UK

**Keywords:** cardiac interoception, exercise pacing, exercise tolerance, rating of perceived exertion

## Abstract

The aim of this study was to examine the role of cardiac interoception on self‐regulated (Experiment 1) and externally prescribed (Experiment 2) exercises. Cardiac interoception was assessed using heartbeat tracking and discrimination tasks in both experiments. Based on heartbeat discrimination performance, participants were partitioned into groups demonstrating GOOD and POOR cardiac interoceptive accuracy. In Experiment 1, 20 participants completed two self‐regulated 20‐min cycling tasks at two intensities (*light* rated physical exertion [RPE on Borg Scale = 10] vs. *hard‐to‐very hard*, RPE = 16). During self‐regulated exercise, the POOR cardiac interoception group showed lower differences in their exercise work rates and physiological responses between *light* and *hard‐to‐very hard* intensity exercises. These differences were partly attributable to a higher work rate over the first 5 min of *light* intensity exercise and a higher initial rate of work in the first min of *hard‐to‐very hard* intensity exercise. In Experiment 2, 15 participants completed an externally prescribed, constant‐load cycling task performed at 80% of the peak power output, to task failure. During externally prescribed exercise, GOOD and POOR groups did not differ in their time‐to‐task failure nor in their physiological and perceptual responses to the exercise. Together these findings demonstrate that individual differences in interoceptive accuracy influence the regulation of self‐paced exercise but do not affect externally prescribed exercise tolerance under constant load.

**Summary**

- This study is the first to examine the effect of cardiac interoception during both externally prescribed constant‐load and self‐regulated exercise.
- In Experiment 1, we demonstrated that those with less accurate cardiac interoceptive task ability produced higher exercise power outputs relative to their physiological capacity and increased physiological strain at *light* self‐regulated exercise intensities, whereas this effect was reversed in the *heavy* self‐regulated intensity.
- In Experiment 2, we observed that individual differences in the cardiac interoception task did not influence exercise tolerance during the constant load task with both GOOD and POOR heartbeat perceivers, demonstrating comparable time‐to‐task failure at 80% of peak power output
- Our findings suggest that cardiac interoception is important in self‐regulated exercise, but does not play a role in the tolerance to externally prescribed constant‐load exercise.

## 1 Introduction

Perceived exertion is a gestalt feeling state reflecting the integrative representation of information from the periphery (e.g., musculoskeletal, cardiovascular and respiratory inputs) alongside central contextual information (including cognition, motivation, attention, and memory) that gives rise to a singular index of perceived physical strain (Hutchinson and Tenenbaum 2006). Borg's rating scale for perceived exertion (RPE, 6–20 scale; G. A. Borg 1982) is a widely used tool for quantifying subjective judgements of perceived exertion during physical exercise with RPE scores known to be sensitive to changes in sensory information consequent to manipulations of internal bodily state or external environment (Brownsberger et al. 2013; De Oliveira Pires and Hammond 2012; Flood, Waldron, and Jeffries 2017; Hampson et al. 2001; Parry and Micklewright 2014). Such studies support the proposal that perceived exertion is an important factor mediating exercise performance, influencing both exercise pacing (de Koning et al. 2011; Tucker 2009) and exercise tolerance (e.g., time‐to‐task failure under constant‐load exercise; Noakes, Snow and Febbraio 2004).

The sensing of internal physiological signals, and their conscious perception, termed as interoception, appears to be important in the perception of exertion (Williamson et al. 1999). Firstly, during the whole‐body exercise, RPE scores increase with somatic perceptions (e.g., muscle pain and breathlessness; Jameson and Ring 2000) that emerge from inputs signalled along interoceptive pathways (Blain et al. 2016; Craig 2003) such as heart rate (*f*_c_) during graded cycling exercise (E. Borg and Kaijser 2006). Secondly, RPE scores during exercise positively correlate with changes in insular cortex activity, a region implicated in the representation and processing of interoceptive signals (Critchley et al. 2004; Williamson et al. 1999). Thirdly, increased attention towards interoceptive cues can augment subjective perceptions of exertion and fatigue (Fillingim and Fine 1986; Pennebaker and Lightner 1980b), whereas decreased attention to (distraction from) interoceptive cues may reduce RPE and increase work capacity during exercise (Mohammadzadeh et al. 2008). Finally, exercise intensity is proposed to influence the relative salience of interoceptive signals during exercise; with interoceptive inputs exerting greater influence over the affective experience at higher exercise intensities (Ekkekakis, Hall, and Petruzzello 2008; Ekkekakis 2009). However, the influence of interoception on both regulation of exercise intensity and exercise tolerance remains unknown. By regulation of exercise intensity, we understand that to mean the control of work rate (or work [in Joules] every second) a person undertakes during an exercise performed on a cycle ergometer. Exercise tolerance on the other hand is classically measured through physiological responses and time‐to‐task‐failure during an exercise of pre‐determined and imposed work rate (usually constant too), which is to be maintained for the longest possible time. Moreover, little is known about the impact of different interoceptive abilities on the relationship between exercise regulation, tolerance and the perception of exertion.

People vary considerably in their capacity to access interoceptive sensations as conscious perceptions (Khalsa et al. 2018; Werner, Duschek and Schandry 2009). This has been shown particularly for the perception of cardiac sensations using tasks that seek to measure perceptual sensitivity to heartbeat sensations during passive resting conditions. In particular, heart beat tracking (HBT; Schandry 1981) and heart beat discrimination tasks (HBD; Whitehead et al. 1977) have been widely used in this context (Critchley and Garfinkel 2018). Performance on the HBT in particular has known sensitivity to other (non‐cardiac) factors that may constrain inference (Desmedt et al. 2020; Ring et al. 2015) yet construct and discriminant validity can be shown (Schulz et al. 2021). To help disentangle individual differences in interoceptive ability, a taxonomy of interoception is proposed that differentiates *accuracy* (an objective measure of a person's performance on interoceptive tasks, such as the HBT and HBD), *confidence* (a person's subjective or self‐evaluative perspective of their interoceptive ability, e.g., rated confidence in task performance), and *awareness* (a metacognitive construct evaluating the correspondence between objective interoceptive accuracy and subjective confidence) (Garfinkel et al. 2015). When applied to the perception of internal states during exercise, this framework can help examine the contribution of interoceptive abilities to both the perception of physiological strain (i.e., RPE) and actual physiological strain (i.e., exercise intensity) during self‐paced and externally prescribed constant‐load exercise where task failure can be examined while controlling for volitional behaviour. However, despite growing appreciation of the potential role of interoception in these processes (Greenhouse‐Tucknott et al. 2021; McMorris, Barwood and Corbett 2018; Wallman‐Jones et al. 2021), to date no previous study has pursued this. Given the hypothesised greater salience of interoceptive signals at higher intensities of exercise (Ekkekakis, Hall, and Petruzzello 2008; Ekkekakis 2009), and the notion that people with greater interoception accuracy are able to direct attention towards interoceptive signals with increased precision (V. L. Ainley et al. 2016), we speculate that individuals with greater interoceptive task accuracy will demonstrate more self‐regulation than those with lower interoceptive task accuracy, particularly at lower levels of exertion. Furthermore, if greater interoceptive task accuracy improves sensitivity to the sensory effects of exercise (i.e., including adverse reactions; Herbert, Ulbrich and Schandry 2007) then, plausibly, individuals with greater (vs. lower) interoceptive task ability will be less tolerant of exercise at any given intensity.

Existing knowledge about the effects of individual differences in cardiac interoception on the subjective experience of physical exercise and associated behavioural regulation (both exercise pacing and time‐to‐task failure) remains limited and somewhat equivocal (Herbert, Ulbrich, and Schandry 2007; Köteles, Éliás et al., 2020; Machado et al. 2019). Therefore, the present study was undertaken to clarify the role of individual differences in cardiac interoceptive ability on the regulation of and tolerance to physical exercise. Thus, two separate experiments were conducted, focussing on (1) exercise regulation (i.e., pacing), and (2) exercise tolerance. For Experiment 1, it was predicted that more accurate heartbeat perception will be associated with lower exercising work rates when expressed relative to individual's aerobic capacity and maximal aerobic power (i.e. in percentage of first and second ventilatory threshold and $\dot{V}$ O_2_peak, respectively—*see* methods) and reduced markers of physiological strain (i.e. heart rate [*f*_c_], oxygen consumption [$\dot{V}$ O_2_] and respiratory exchange ratio [RER]) for a given perceived exercise intensity (RPE level). Two different perceived exertion levels (*light* RPE10 vs. *hard‐to‐very hard* RPE16) were given to the participants to regulate to, thus permitting the differences in exercise self‐regulation across two different perceptual exercise intensities to be examined in people with high versus low interoceptive abilities (Ekkekakis, Hall, and Petruzzello 2008; Ekkekakis 2009). For Experiment 2, it was predicted that those with greater cardiac interoceptive task accuracy would result in reduced performance in a constant‐load time to failure task due to enhanced sensitivity to interoceptive signalling (Herbert, Ulbrich, and Schandry 2007). Finally, measures of cardiac interoceptive task confidence and awareness were included alongside cardiac interoceptive task accuracy to quantify the relative influence of these additional dimensions on exercise behaviour (i.e., exercise regulation for *Experiment 1* and exercise termination for *Experiment 2*).

## 2 Methods

### 2.1 Procedures Common to Both Experiments

Participants were recruited from flyers and were screened in‐person based on medical history and provided written informed consent prior to enrolment. All participants were physically active and accustomed to exhaustive exercise. Experimental procedures were approved by the local Research Ethics and Governance Committee and conducted in accordance with the guidelines outlined in the 2024 Declaration of Helsinki, except for prior registration in a database. Participants were required to refrain from strenuous exercise (48 h), alcohol (24 h), and caffeine consumption (12 h) before each visit and asked to arrive at the laboratory in a rested and hydrated state, at least 2 h postprandial. Each participant undertook testing at the same time of day ±1 h, to minimise the influence of circadian rhythm on cycling performance (Teo, Newton, and McGuigan 2011) with a minimum 48 h between tests.

Sample sizes for a within–between interaction from the *F* test family were determined by power analysis (G*Power 3.1, Erdfelder, Faul, and Buchner 1996; *α* = 0.05, *β* = 0.20, Number of groups = 2, Number of measures = 5, Correlation among measures = 0.5, *ε* = 1) using preliminary data from our lab, with an effect size (*f* = 0.625) derived from differences in aerobic fitness between good and poor heartbeat perceivers (HBD accuracy). This effect size is larger than those reported for comparable studies using HBT accuracy (e.g., Herbert, Ulbrich, and Schandry 2007; Machado et al. 2019) but smaller than that reported for physiological indices of cardiac interoceptive processing (Perakakis et al. 2017). The strength of this effect size, compared with HBT accuracy, presumably reflects the greater robustness of the HBD task to temporal estimation biases (Desmedt et al. 2020). For Experiment 2, the sample size calculation for a difference in times‐to‐task failure between the two independent groups (G*Power 3.1, Erdfelder, Faul, and Buchner 1996; *α* = 0.05, *β* = 0.20, 0.8 effect size, allocation ratio of 1:1; two‐tailed *t*‐test) led to the recruitment of 26 participants.

#### 2.1.1 Interoception Tasks

Cardiac interoception was assessed according to the methods described by Garfinkel and colleagues (2015), which included both HBT and HBD tasks, conducted using a proprietary script with a deployable graphic user interface (MATLAB Compiler 2016b; The MathWorks Inc., Natick, MA, USA). Pulses were detected using Nonin OEM development kit (XPOD OEM III, Nonin Medical Inc., Plymouth, MN, USA) connected to a personal computer (Hewlett Packard, Palo Alto, USA). A soft sensor (Murphy et al. 2019) was attached to the index finger of the non‐dominant hand with the arm resting on a foam mat. Participants were seated throughout the assessment with watches and jewellery removed and minimal ambient noise. Participants completed the HBT task first. For the HBT, participants completed one familiarisation trial (20 s) and six experimental trials of different durations (25, 30, 35, 40, 45 and 50 s) presented in a randomised order. For each trial, participants reported the number of perceived heartbeats and provided a confidence rating of the perceived accuracy of their perception, denoted by a single vertical pen stroke on a visual analogue scale. For HBD, participants made synchronicity judgements between a series of audible tones (440 Hz, 100 ms duration) and the timing of their heartbeat. Reporting of these judgements was made through simple ‘in sync’ or ‘out of sync’ responses and a subsequent rating of confidence. For synchronous conditions, audible tones were presented 250 ms after the R‐wave; non‐synchronous conditions audible tones were presented 550‐ms after the R‐wave (Betka et al. 2020; Wiens and Palmer 2001). In total, testing comprised one familiarisation (20‐s duration) and 20 experimental trials (10 synchronous, 10 asynchronous; presented in a randomised order), each consisting of 10 audible tones per trial.

Accuracy for HBT was determined by comparing differences between the number of heartbeats reported and recorded in each trial and performance quantified using the equation described by Hart et al. (2013). A singular metric for HBT accuracy was obtained from the average of the accuracy scores across the six trials. For HBD, accuracy was assessed using the number of correct responses relative to the total number of trials. Confidence measures were obtained separately for each task by averaging the scores across the trials. Awareness in the HBT task was calculated using Pearson's correlation coefficient (*r*) comparing the accuracy and confidence scores for each individual (Garfinkel et al. 2015). For the HBD task, awareness was evaluated as the area under the curve obtained from a receiver operator characteristic (ROC) analysis of responses (correct/incorrect) inputted against confidence scores (Green and Swets 1966).

#### 2.1.2 Exercise Testing

All cycling tests were performed on a cycle ergometer (high‐performance ergometer with Rohloff Gear Hub, SRM, GmbH, J $\ddot{u}$ lich, Germany). Participants were instrumented with a heart rate monitor (A300 Fitness Watch, Polar Electro Oyo, Temple, Finland) and face mask connected to an online gas‐analysis system MetaLyzer 3B, Cortex, Germany). Before each test, both cycle ergometer and online gas‐analysis system were calibrated according to manufacturer's instructions. Each experimental trial was preceded by 3‐min resting sample of expired gases and *f*_c_ before commencing a 5‐min warm‐up at 60 W (pedalling frequency kept at 80 revolution per minute [rev·min^−1^]) followed by a further 2 min of rest. Participants were required to maintain a cadence of 80 rev min^−1^ during each test.

Participant's aerobic fitness was determined prior to each experiment using a graded exercise test (GXT). Power output (PO) was increased by 20 W every minute with initial PO estimated based on the experimenter's judgement of the participants' training history and status (60–160 W). Testing was terminated when cadence dropped below 75 rev min^−1^ for more than 5 s despite strong verbal encouragement (Ross 2003). For expired gases, breath‐by‐breath data were converted to second‐by‐second (MetaLyzer Studio, Cortex, Germany) and analysed across rolling 30‐s averages. For a measure of participant's aerobic capacity, oxygen consumption at the first ventilatory threshold and respiratory compensation point ($\dot{V}$ O_2_ at VT_1_ and VT_2_, respectively) were identified using the method of ventilatory equivalents (Bhambhani and Singh 1985; Dekerle et al. 2003). VT_1_ represents the first increase in minute ventilation (V̇_E_) proportional to the increase in CO_2_ output (V̇CO_2_). As a result, the ventilatory equivalent for oxygen (V̇_E_/V̇O_2_) increases with no change in the ventilatory equivalent for carbon dioxide (V̇_E_/V̇CO_2_). VT_2_ corresponds to the subsequent additional hyperventilation where V̇_E_ and both ventilatory equivalents show a marked increase, whereas end‐tidal partial pressure of carbon dioxide (P_ET_CO_2_) starts to decrease. Identification of these thresholds were made by the lead investigator and corroborated by an independent observer. Linear interpolations from the PO—$\dot{V}$ O_2_ relationship provided PO estimates (i.e. PO_VT1_ and PO_VT2_). For a measure of participant's maximal aerobic power, peak PO (PO_peak_) and $\dot{V}$ O_2_ ($\dot{V}$ O_2peak_) were defined as the highest 30‐s average value during the GXT (Ross 2003). These determinations of both aerobic capacity and maximal aerobic power enabled absolute PO (PO_abs_; in Watts) recorded for the self‐paced trials in Experiment 1 and for the constant‐load trials in Experiment 2 to be expressed relative to individual's aerobic fitness (i.e., as a percentage of the PO recorded at VT_1_ (%PO_VT1_), VT_2_ (%PO_VT2_), and PO_peak_ (%PO_peak_).

Respiratory gas exchange and heart rate (*f*_*c*_) was also recorded throughout all exercise tasks. For post‐hoc analyses, rolling 30 s averages were used. Respiratory variables of interest were oxygen consumption ($\dot{V}$ O_2_) and respiratory exchange ratio (RER, i.e., $\dot{V}$ O_2_/$\dot{V}$ CO_2_) for insights into energy expenditure and substrate utilisation.

RPE was assessed using the Borg RPE scale, a single 15‐item scale where perceived exertion was rated between 6 (*no exertion*) and 20 (*maximal exertion*), with descriptive terms for exertion provided at negative integers. Standardised instructions were presented to participants prior to all exercise tasks. Instructions were in accordance with the authors' recommendations (G. A. Borg 1998), encouraging participants to concentrate on their current overall experience of physical exertion rather than focussing on specific, localised sensations (e.g., local muscle discomfort).

### 2.2 Experiment 1

Twenty‐four individuals (14 males, 10 females; 23 ± 3 years, 172 ± 9 cm, 68.7 ± 10.3 kg) completed this experiment. Participants visited the laboratory on three occasions. During the first visit, participants completed the cardiac interoception task and GXT. In the subsequent visits, participants completed a fixed‐RPE task at either *light* (RPE10) or *hard‐to‐very hard* (RPE16) ratings of exertion, with the order of these trials randomised between participants. For these tasks, the cycle ergometer was set to isokinetic mode, maintaining a pedalling cadence of 80 rev min^−1^ with participants required to self‐regulate exercise intensity by altering the effort produced through the pedals. Prior to the fixed‐RPE tasks, participants were reminded of the procedures and requirements of the exercise task, including description of the Borg scale and the required level of exertion corresponding to either RPE10 or RPE16. RPE was obtained every 5 min to assess adherence to the task constraints. Scripted verbal feedback was also provided every 2 min to remind participants to maintain the desired RPE.

Physiological data (expired gases, *f*_c_) were averaged over 60 s epochs for the first 5‐min and 5‐min epochs from minute 5 onward. This approach was used to reflect the changing nature of V̇O_2_ kinetics during the early stages of exercise, whereby dynamic changes in V̇O_2_ responses are observed in the first 3–5 min of exercise are followed by a more stable (steady‐state or slow) component thereafter (A. M. Jones and Burnley 2009). Epochs are reported according to the final minute of the recording (e.g., minute 15 refers to data averaged between minutes 10–15).

### 2.3 Experiment 2

Twenty‐six participants (13 males, 13 females; 24 ± 3 years; 173 ± 9 cm; 69.7 ± 12.0 kg) voluntarily completed this experiment, including eight volunteers who participated in Experiment 1. They visited the laboratory on two occasions. During the first visit, participants completed the cardiac interoception task and GXT. In the second visit, participants completed a constant‐load exercise task set at 80% of PO_peak_ to task failure. The work rate of 80% PO_peak_ was chosen for its closeness to the upper limit of the heavy intensity domain (Dekerle et al. 2003; Olson, Tracy, and Dengel 2009). RPE was obtained from the participants in the final 15 s of each minute for the first 4 min of the test and again at the end of the test. Physiological data (expired gases, *f*_c_) were averaged over 60 s epochs for the first 4‐min and over final minute prior to task failure. Termination criteria for the test was defined as a reduction in cadence below 70 rev·min^−1^ for more than 10 s. Participants were excluded from the final analysis if they failed to achieve a minimum performance time of 4 min before exercise termination or were identified as being extreme outliers (*z*‐score deviation from the mean > 3.29 [99.9th percentile]).

### 2.4 Statistical Analysis

For between‐groups analyses, participants were retrospectively allocated as good (GOOD) or poor (POOR) heartbeat perceivers according to a whole‐group median demarcation of the HBD accuracy data (POOR ≤ $\overset{∼}{x}$ < GOOD). HBD accuracy was selected as the HBD task is considered more robust to temporal estimation bias compared with HBT (Phillips et al. 1999), with accuracy representing the ‘core perceptual construct’ underpinning both confidence and awareness (Garfinkel et al. 2015). The groups were further differentiated by application of 95% confidence intervals (95% CI) to each group, removing participants with accuracy scores located between the sample median value and the adjacent 95% CI boundary. This approach was employed to minimise potential limitations associated with dichotomising samples based on continuous variables (Altman and Royston 2006). This approach led to samples sizes of 9 (GOOD) and 11 (POOR) for experiment 1 and 7 (GOOD) and 8 (POOR) for experiment 2.

Statistical analysis was completed using SPSS 25 (IBM, New York, USA). Assumptions of normality and sphericity were examined using Shapiro–Wilk's test and Mauchly's test, respectively. Group characteristics for stature, mass, age, aerobic fitness, and cardiac interoception were examined using independent samples *t*‐tests, which was also applied to the time‐to‐task failure in Experiment 2. Two‐way mixed‐methods ANOVAs were used to compare between GROUP (2: GOOD, POOR) and across TIME (3 [minutes 10, 15, 20]), and between GROUP (2: GOOD, POOR) and TIME (5 [minutes 1–5]) in Experiment 1. In Experiment 2, between GROUP (2: GOOD, POOR) and across TIME (5 [minutes 1, 2, 3, 4, End]) comparisons were examined. Greenhouse–Geisser corrections were applied if Mauchly's value was ≤ 0.75 with Huynh–Feldt correction used when Mauchly's value was > 0.75. For all ANOVAs, *post‐hoc* analysis were performed using Bonferroni‐corrected pairwise comparisons. For exploratory correlations, participants omitted based on the 95% CI group criterion were reincluded. Correlations (Pearson's *r* or Kendal's τ_b_) were performed comparing measures of cardiac interoception (HBT, HBD; accuracy, confidence and awareness) the change in PO from RPE10 to RPE16 conditions (trial mean, %PO_peak_) (Experiment 1) and time‐to‐task failure (Experiment 2). Parametric data are presented as mean ± SD and non‐parametric data are presented as median (lower quartile—upper quartile). Effect sizes were established using $\eta_{p}^{2}$ for main effects in the ANOVAs and Hedges' *g* for *t*‐tests.

## 3 Results

### 3.1 Experiment 1

#### 3.1.1 Group Allocation

Application of the group allocation criterion resulted in the removal of four participants from the between‐groups analyses (two participants; each from GOOD and POOR). Group characteristics are shown in Table S1. GOOD were more accurate in their HBD as a result of the grouping criteria (*p* < 0.01) and demonstrated greater confidence in HBT compared with POOR (*p* = 0.02). HBD awareness was marginally non‐significant between groups (*p* = 0.06, *g* = 0.95). Differences in aerobic fitness were also found to be non‐significant between the two groups despite large effect sizes for PO_VT1_ (*p* = 0.06, *g* = 0.91), PO_VT2_ (*p* = 0.06, *g* = 0.86) and PO_peak_ (*p* = 0.06, *g* = 0.87).

Reported RPE values during RPE10 and RPE16 were not significantly different between the two groups (*p* > 0.15) and matched the target RPE at all timepoints, except for minute 5 in the RPE16 condition (GOOD: 14 [13–15]; POOR: 15 [15–15.5]) as shown in Table S7.

**FIGURE 1:**
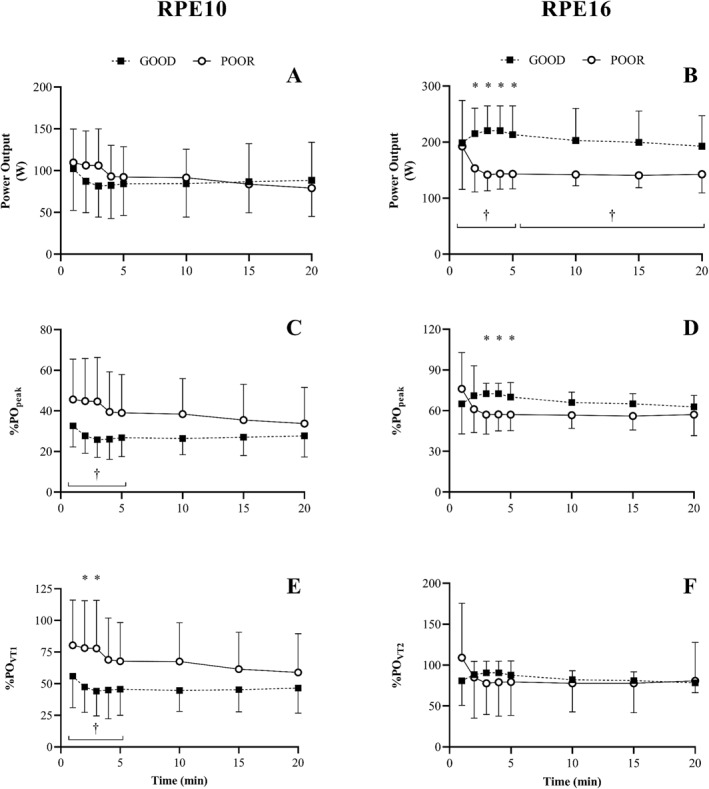
Power output (PO) responses during the 20‐min self‐regulated exercise task at light rating of perceived exertion (RPE10; left) and hard‐to‐very hard rating of perceived exertion (RPE16; right) for both good (GOOD) and poor (POOR) heartbeat perceivers. PO represented in absolute terms (A, B) normalised to the participants peak PO obtained in the graded exercise test (C, D), and normalised to participants PO at the first (E) and second (F) ventilatory threshold. ^†^Denotes a significant GROUP effect. *Denotes significant *post hoc* differences between groups for the GROUP × TIME interaction effect. Main effects for TIME and GROUP × TIME omitted for clarity.

Summary tables of the ANOVA statistics for Experiment 1 can be found in Table S3 and S4. Analysis of the PO data for RPE10 revealed significant TIME effects for all measures of PO (i.e., PO_abs_, *F* = 4.8, *p* = 0.02, *η*_p2_ = 0.21, %Trial_mean_ *F* = 5.8, *p* = 0.01, *η*_p2_ = 0.24, %PO_VT1_ *F* = 4.0, *p* = 0.03, *η*_p2_ = 0.18, %PO_peak_ *F* = 3.7, *p* = 0.04, *η*_p2_ = 0.17) over the first 5 min but not for minutes 10–20 (Figure 1A,C,E). *Post hoc* analysis revealed that PO was lower at minute 3 compared with minute 1 (%PO_peak_, *p* = 0.04) and lower at minute 4 compared to minute 1 (%Trial_mean_, *p* = 0.01). Significant GROUP effects were also found over the first 5 min for %Trial_mean_ (*F* = 6.2, *p* = 0.02, *η*_p2_ = 0.26, %PO_VT1_) (*F* = 4.5, *p* = 0.047, *η*_p2_ = 0.20, GOOD: 48 ± 28; POOR: 75 ± 28) and %PO_peak_ (*F* = 4.6, *p* = 0.046, *η*_p2_ = 0.20, GOOD: 28 ± 14; POOR: 43 ± 17). A significant GROUP effect was found for %Trial_mean_ at minutes 10–20 (*F* = 6.2, *p* = 0.02, *η*_p2_ = 0.02). Interaction effects were only found at minutes 10–20 in all variables but were not significant following post hoc analysis.

For RPE16, a significant effect of GROUP was found for PO_abs_ for both minutes 1–5 (*F* = 9.7, *p* = 0.01, *η*_p2_ = 0.35, GOOD: 214 ± 38 W, POOR: 156 ± 46 W) and minutes 10–20 (*F* = 9.5, *p* = 0.01, *η*_p2_ = 0.34, GOOD: 198 ± 37 W, POOR: 142 ± 45 W) with no significant GROUP effects found for other measures of PO. No significant TIME effects were found for minutes 1–5 or 10–20. However, significant interaction effects were found for all measures of PO over the first 5 min (i.e., PO_abs_, *F* = 5.8, *p* = 0.02, *η*_p2_ = 0.24, %Trial_mean_ *F* = 5.6, *p* = 0.02, *η*_p2_ = 0.24, %PO_VT2_ *F* = 5.9, *p* = 0.02, *η*_p2_ = 0.26, %PO_peak_, *F* = 6.7, *p* = 0.01, *η*_p2_ = 0.27) but not for minutes 10–20, suggesting that the two groups adopted differing pacing strategies over the initial 5 min of the RPE16 task (Figure 1B,D,F). *Post hoc* analysis revealed significant differences between the two groups for minute 2 (PO_abs_), minute 3 (PO_abs_, %Trial_mean_, %PO_peak_), minute 4 (PO_abs_, %Trial_mean_, %PO_peak_), and minute 5 (PO_abs_, %PO_peak_).

**FIGURE 2:**
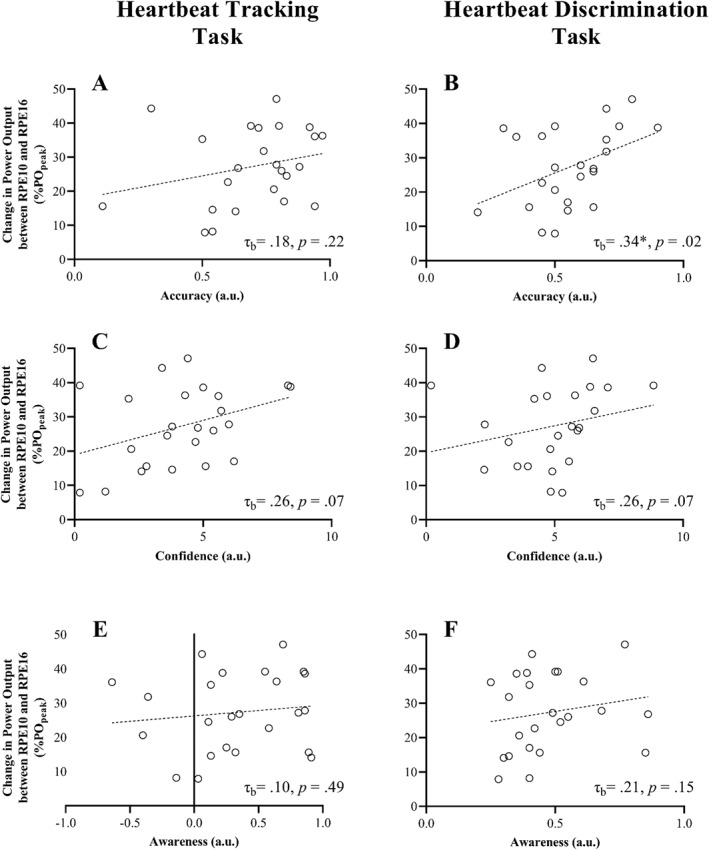
Relationship between changes in average power output (normalised to %PO_peak_) from RPE10 to RPE16 with interoceptive characteristics (HBT, left; HBD, right) for: Accuracy (A, B), Confidence (C, D) and Awareness (E, F). τ_b_ = Kendal's rank correlation coefficient. *Significant correlation (*p* < 0.05). Trendlines included for visual purposes only.

In exploratory analyses, we compared changes in PO from RPE10 to RPE16 with measures of cardiac interoception. The HBD accuracy was found to correlate positively with the change in PO between the two fixed‐RPE conditions (Figure 2; τ_b(24)_ = 0.30, *p* = 0.048), suggesting that more accurate HBD perception was associated with a greater change in PO between RPE10 and RPE16. No significant correlations were found for the other measures of cardiac interoception.

**FIGURE 3:**
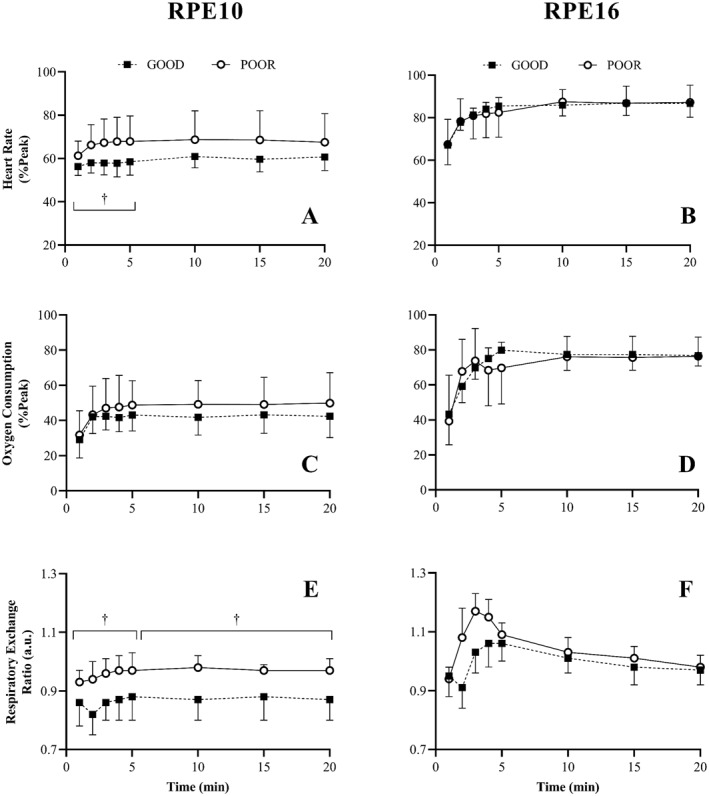
Heart rate (A, B), oxygen consumption (C, D) and respiratory exchange ratio (E, F) responses during the 20‐min self‐regulated exercise task at light rating of perceived exertion (RPE10; left) and hard‐to‐very hard rating of perceived exertion (RPE16; right) for both good (GOOD) and poor (POOR) heartbeat perceivers. PO represented in absolute terms (A, B) normalised to the participants peak PO obtained in the graded exercise test (C, D), and normalised to participants PO at the first (E) and second (F) ventilatory threshold. ^†^Denotes a significant GROUP effect. Main effects for TIME omitted for clarity.

Summary tables of the ANOVA results for the cardiorespiratory responses can be found in the Table S5. Cardiorespiratory responses to the fixed‐RPE tasks are shown in Figure 3. For RPE10, post hoc analysis of the significant TIME effects revealed that *f*_c_, $\dot{V}$ O_2_, and RER increased significantly over the first 5 min (i.e., *f*_c_, *F* = 6.9, *p* = 0.01, *η*_p2_ = 0.28, $\dot{V}$ O_2_ *F* = 16.5, *p* < 0.01, *η*_p2_ = 0.48, RER *F* = 6.5, *p* < 0.01, *η*_p2_ = 0.24) with no significant change in values for minutes 10–20 (*p* values > 0.49). Significant GROUP effects were observed over the first 5 min for both *f*_c_ (*F* = 5.6, *p* = 0.03, *η*_p2_ = 0.24, GOOD: 58 ± 3, POOR: 66 ± 2) and RER (*F* = 16.7, *p* < 0.01, *η*_p2_ = 0.48, GOOD: 0.86 ± 0.07 a.u., POOR: 0.96 ± 0.06 a.u) with significant GROUP effects also evident for RER for minutes 10–20 (*F* = 16.8, *p* < 0.01, *η*_p2_ = 0.48, GOOD: 0.87 ± 0.07 a.u., POOR: 0.97 ± 0.04 a.u.). No differences were found for $\dot{V}$ O_2_ between the two groups for either the first 5 min (*p* = 0.45, $\eta_{p}^{2}$ = 0.03) or minutes 10–20 (*p* = 0.26, $\eta_{p}^{2}$ = 0.07). Only RER at RPE 10 was found to interact significantly (*F* = 16.8, *p* < 0.01, *η*_p2_ = 0.48.). The RER of the POOR group significantly decreased from minutes 10 to 15 only. No significant change was found in the GOOD group over minutes 10–20.

For RPE16, TIME effects were evident for *f*_c_, $\dot{V}$ O_2_, and RER, which all increased significantly over the first 5 min (i.e., *f*_c_, *F* = 364.6, *p* < .01, *η*_p2_ = .95, $\dot{V}$ O_2_ *F* = 213.4, *p* < .01, *η*_p2_ = .92, RER *F* = 186.6, *p* < .01, *η*_p2_ = .91). RER was also found to increase over minutes 10–20 (*F* = 10.1, *p* < .01, *η*_p2_ = .36). No significant GROUP or interaction effect were found for RPE16.

### 3.2 Experiment 2

Based on the exclusion criteria for task performance, three participants were removed from the final dataset with a further eight participants excluded from the between‐group analysis because of the interoception group membership criterion. Demographic data for the two groups are shown in Table S2. Stature (*p* = 0.02) and body mass (*p* = 0.03) were significantly greater for GOOD, likely reflecting differences in gender distributions of the two groups. No differences were observed between groups with respect to age (*p* = 0.76). Analysis of cardiac interoception revealed significant differences in HBD accuracy only (*p* < 0.01). GOOD produced significantly greater PO's at VT_1_ (*p* = 0.04), VT_2_ (*p* = 0.03) and peak (*p* = 0.03) compared with POOR. No significant differences were found for any of the other measured variables, despite large effect sizes being evident for the $\dot{V}$ O_2_ values at VT_1_ (*g* = 0.85), VT_2_ (*g* = 0.86), and peak (*g* = 0.89) between the groups.

Average PO during the constant‐load task was 250 ± 62 W for GOOD and 191 ± 34 W for POOR, corresponding to 98 ± 10% and 104 ± 11 %PO_VT2_, respectively (*p* = 0.26, *g* = 0.54). Time‐to‐task failure were also not different between the two groups (Figure S1; *p* = 0.29, *g* = 0.19). Considering the differences in gender distribution of the two groups (Table S2), exploratory analysis comparing male (468 ± 118 s) and female (405 ± 94 s) participants was performed on the time‐to‐task failure but again no differences were found (*p* = 0.27, *g* = 0.56). Correlational analysis comparing time‐to‐task failure with measures of relative exercise intensity, described in terms of %PO_VT1_ (*r*_(15)_ = −0.475, *p* = 0.07, 95% CI = [−0.04, 22]), %PO_VT2_ (*r*_(15)_ = −0.311, *p* = 0.26, 95% CI = [−0.00, 20]), and %PO_peak_ (*r*_(15)_ = −0.496, *p* = 0.06, 95% CI = [−0.02, 16]) indicated that performance was not associated with differences in the relative intensity of the constant‐load task.

**FIGURE 4:**
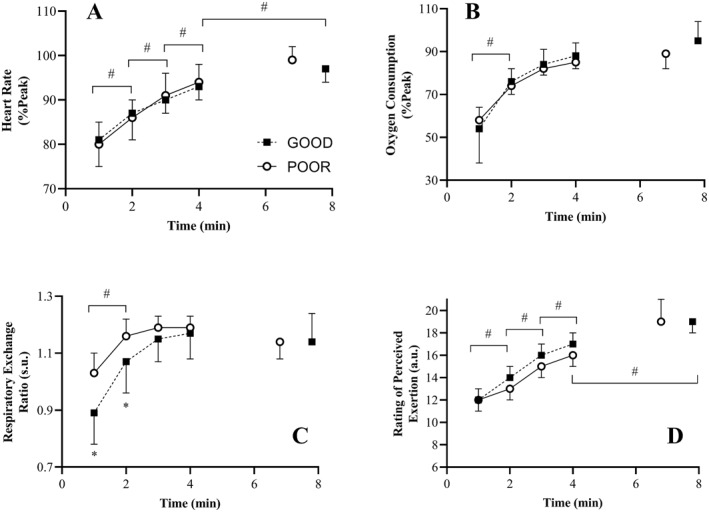
Differences between good (GOOD) and poor (POOR) heartbeat perceivers on the physiological and perceptual responses during a constant load cycling task at 80% PO_peak_. (A) Oxygen consumption (%Peak). (B) Respiratory exchange ratio. (C) Heart rate (%Peak). (D) Rating of perceived exertion. Data presented as mean ± SD. $\dot{V}$ O_2_ and *f*_c_ data normalised based peak values obtained from a graded exercise test. s.u. = standardised units. a.u. = arbitrary units. ^#^Significant *post hoc* differences between timepoints for the effect of TIME (*p* < 0.05). *Significant *post hoc* differences between groups for the GROUP × TIME interaction effect.

Cardiorespiratory measures are shown in Figure 4 and ANOVA results are shown in Table S6. A significant TIME effect was observed for *f*_c_ , RER, $\dot{V}$ O_2_ (*f*_c_, *F* = 195.6, *p* < 0.01, *η*_p2_ = 0.94, $\dot{V}$ O_2_ *F* = 113.7, *p* < 0.01, *η*_p2_ = 0.90, RER *F* = 69.7, *p* < 0.01, *η*_p2_ = 0.84). *Post hoc* analysis for $\dot{V}$ O_2_ indicated differences between minutes 1 and 2 only (*p* < 0.01) with differences in RER evident between minutes 1 and 2 (*p* < 0.01) and minutes 2 and 3 (*p* < 0.01). For *f*_c_, significant differences were found between all timepoints (*p*'s < 0.01). No significant GROUP effects were found for any measure (*p* ≥ 0.06, *η*_p2_ ≤ 0.25). An interaction effect was found for RER only (*F* = 5.7, *p* = 0.02, *η*_p2_ = 0.28) with lower RER in GOOD at minutes 1 and 2 (*p* < 0.05).

Analysis of the data indicated that RPE scores increased significantly over TIME (*F* = 106.478, *p* < 0.01, *η*_p2_ = 0.89) but were not significantly different for either the main effect of GROUP (*F* = 3.020, *p* = 0.11, *η*_p2_ = 0.19) or the interaction effect (*F* = 0.641, *p* = 0.59, *η*_p2_ = 0.05). Post hoc analysis of the TIME effects revealed significant differences between all timepoints (*p* < 0.01).

Relationships between time‐to‐task failure and measures of cardiac interoception were non‐significant including: HBT accuracy (*r*_(23)_ = 0.38, *p* = 0.08, 95% CI = [0.05, 0.63]), HBT confidence (*r*_*s*(23)_ = 0.06, *p* = 0.80, 95% CI = [−0.39, 0.50]), HBT awareness (*r*_(23)_ = 0.15, *p* = 0.51, 95% CI = [−0.30, 0.51]), HBD accuracy (*r*_(23)_ = 0.27, *p* = 0.22, 95% CI = [−0.21, 0.61]), HBD confidence (*r*_(23)_ = 0.27, *p* = 0.22, 95% CI = [−0.26, 0.62]) and HBD awareness (*r*_(23)_ = −0.01, *p* = 0.95, 95% CI = [−0.51, 0.47]).

## 4 Discussion

The aim of the present study was to examine the influence of cardiac interoception on the regulation and tolerance of physical work during exercise. In Experiment 1, we demonstrated that those with less accurate cardiac interoceptive task ability produced higher exercise power outputs relative to their physiological capacity and increased physiological strain at *light* self‐regulated exercise intensities while this effect was reversed in the *heavy* self‐regulated intensity. Furthermore, these different interoceptive HBD task abilities were associated with the use of different pacing strategies over the first 5 min between the light and hard exercise conditions: no differences in pacing strategies were apparent in the light condition. However, in the heavy condition, participants with lower cardiac interoceptive task accuracy had a greater initial power output in the first minute then decreased their power output during the first 5 min, whereas participants with greater cardiac interoceptive task accuracy demonstrated small increases power output (see Figure 1A, B). In Experiment 2, we observed that individual differences in the cardiac interoception task did not influence exercise tolerance during the constant load task, with both GOOD and POOR heartbeat perceivers demonstrating comparable time‐to‐task failure at 80% of peak power output. Furthermore, these groups did not differ significantly in their physiological data (e.g., *f*_c_, $\dot{V}$ O_2_) or RPE. Taken together, these findings support our prediction that cardiac interoception is important in self‐regulated exercise. However, we found no evidence supporting the hypothesis that cardiac interoception influences tolerance to externally prescribed constant‐load exercise.

During exercise, interoception is considered to be important in the regulation of ongoing exercise intensity, including decisions related to task‐ending (McMorris, Barwood, and Corbett 2018; McMorris 2020). Previous research demonstrates that greater sensitivity (Herbert, Ulbrich, and Schandry 2007) or attention (Fillingim and Fine 1986; Pennebaker and Lightner 1980b) to interoceptive feedback is associated with reductions in the work exerted by participants during self‐paced exercise. In our study, the inclusion of two distinct exercise intensity domains provides a more nuanced examination of the role of cardiac interoceptive accuracy. Previously, it has been found that decreases in work rates at self‐selected intensities (i.e., preferred exercise intensity) relates to either greater interoceptive accuracy or increased interoceptive attention (Herbert, Ulbrich, and Schandry 2007; Pennebaker and Lightner 1980a). However, this effect was not observed in our data. Instead, when examined according to relative work rate and cardiorespiratory measures, observed differences between the two groups indicates that people with lower levels of interoceptive task accuracy exert themselves to a higher level of physiological strain for the *light* intensity exercise but not for the heavier intensity exercise. At *light* intensities, these individuals required larger changes in physiological strain than those with greater interoceptive task accuracy presumably to drive interoceptive feedback to such an extent to provide sufficient confidence in their estimate of perceived exertion. At heavier intensities, this process resulted in different pacing strategies. Individuals with lower interoceptive task accuracy were found to adopt a ‘drive‐to‐awareness’ pacing strategy at the start, with power output in the first minute of the *heavy* intensity exercise condition being one third greater than at the fifth minute, and one third greater than their average power output for the whole condition, despite the participants with greater interoceptive task accuracy producing more absolute power. This ‘drive‐to‐awareness’ pacing strategy for the lower interoceptive accuracy group suggests less initial sensitivity to interoceptive information signals. Potentially that account accords with a predictive coding framework applied to exertional fatigue (Greenhouse‐Tucknott et al. 2021); a behavioural strategy, such as early ‘drive‐to‐awareness’ pacing, can be selected to increase the certainty of perceived exertion through large and immediate changes in interoceptive signalling, facilitating a greater confidence (precision‐weighting) in the perception of changes in bodily states. In contrast, individuals with greater interoceptive accuracy are able to regulate exercise more immediately through predictive ‘top‐down’ precision‐weighting achieved through attentional processes that may increase the likelihood of ‘bottom‐up’ prediction‐error signals ascending for conscious appraisal (Greenhouse‐Tucknott et al. 2021).

By contrast, in Experiment 2 we showed that cardiac interoceptive task accuracy did not influence time‐to‐task failure and the two interoceptive groups were similar in terms of physiological strain and their subjective rating of perceived exertion. This finding is comparable with earlier studies that report no effect of individual differences in cardioceptive accuracy on performance in a graded exercise task, nor the ratings of perceived exertion across three physical loads (Machado et al. 2019; Köteles, Teufel, Körmendi, Ferentzi and Szemerszky 2020). Importantly, the effects observed in these studies were all obtained using externally prescribed exercise tasks where autonomy of the management of allostatic demands (through adjustments in muscular work) are constrained and exteroceptive in origin. The intensity‐dependent effects in Experiment 1 extend previous observations, including the finding that cardiac interoceptive accuracy is moderately correlated with more accurate reproduction of heart rates for low (*r*_s_ = 0.32), but not moderate or high, exercise intensities (Köteles, Éliás et al., 2020). These effects can be consolidated when we consider the effect of arousal and attention on interoceptive accuracy: Cardiac interoceptive accuracy transiently improves with the augmentation of physiological arousal, either immediately following exercise (G. Jones and Hollandsworth 1981) or through pharmacological intervention (Khalsa et al. 2020; Khalsa et al. 2009). However, these positive effects appear greater in individuals demonstrating low cardiac interoceptive accuracy at rest, with negligible changes evident in good perceivers (G. Jones and Hollandsworth 1981), suggesting that there is a generalised convergence in cardiac interoceptive accuracy with increasing arousal. Therefore, it is predicted that the influence of cardiac interoceptive accuracy on factors regulating exercise intensity related to trait interoceptive sensitivity may progressively diminish with increasing physiological arousal.

Cardiac interoceptive ability is shown here to play an important role in shaping the regulation of exercise behaviour. However, there are discrepancies between our results and earlier findings. There are, for example, differences in the exercise behaviour data from this study when compared to previous reports (Herbert, Ulbrich, and Schandry 2007; Pennebaker and Lightner 1980a). These may plausibly reflect the differing intentions of the respective exercise protocols, specifically the use of preferred exercise intensity compared with fixed‐RPE. Preferred exercise intensity, as used in previous studies cited above, is likely to reflect the optimisation of a person's core affective experience during exercise (Oliveira, Deslandes, and Santos 2015), a measure that describes the integration of hedonic and arousal components (Russell 2009), whereas RPE more strongly reflects a generalised perception of somatic stress (G. A. Borg 1982). Although affect and exertion emerge, in part, from the processing of afferent inputs within shared cortical networks (Critchley and Nagai 2012; Williams, Hoffman, and Clark 2014), previous studies demonstrate that these two perceptual constructs are dissociable during exercise (Eston et al. 2012; Hamlyn‐Williams, Freeman, and Parfitt 2014; Renfree et al. 2012; Sheppard and Parfitt 2008). This potential division is supported by an observed positive association between measures of cardiac interoceptive accuracy (HBT accuracy) and perceived arousal, but not with affective valence or RPE during exercise (Köteles, Teufel, et al. 2020). Recently, the metacognitive measure of cardiac interoceptive *awareness* was shown to predict the experience of fatigue during isometric knee extension exercise (Greenhouse‐Tucknott et al. 2022). Collectively, the findings from these studies supports the role of interoception in the experience of various feeling states experienced during exercise (e.g., perceived exertion, arousal, affect, and fatigue). However, additional research is needed to further clarify how different dimensions of our interoceptive experience (e.g., accuracy, sensibility, awareness) influence the emergence these different exercise‐induced feeling states and their impact on exercise regulation.

### 4.1 Limitations

The application of different exercise tasks and exercise intensities across the two experiments of this study provided a novel approach to investigate the factors involved in regulating exercise behaviour; however, they are not without limitation. The use of a causal‐comparative design does not allow us to rule out a factor other than interoception explaining group differences in exercise behaviour. The sample size of both experiments was modest, and the findings, particularly explanatory correlations, may be subject to issues of statistical power. For Experiment 2, the use of a singular exercise intensity precludes interpretation of intensity‐dependent effects when considering externally prescribed exercise tasks. For Experiment 1, constraining exercise intensity based on single‐item measures of RPE, as determined using the Borg scale, may be limited in application as these measures represent super‐ordinate constructs that may diverge from changes in underlying physiological responses (Venhorst, Micklewright and Noakes 2018). Consequently, single‐item RPE measures may be deficient in their capacity to distinguish between qualitatively distinct variations of perceived exertion which might occur across different physiological systems, reducing certainty of perceptual information driving exercise behaviour under different contexts. Finally, the influence of interoception in this study can only be considered in the context of the measurement of heartbeat perception at rest and may not reflect (1) the concurrent influence of other interoceptive axes (e.g., respiratory; Garfinkel et al. 2016); or (2) different interoceptive experiences during exercise (Köteles, Éliás et al., 2020). There is some contention in the literature about the heartbeat tracking task validity in indexing an individual's psychological representation of their cardioceptive sensations (Corneille et al. 2020; V. Ainley et al. 2020; Zimprich, Nusser, and Pollatos 2020). The core issues argued is that performance accuracy on the heartbeat tracking task is influenced by response bias and other non‐interoceptive (expectations and estimations) processes. However, a ‘pure’ interoceptive cardioceptive task that can be used dynamically and isolate sensory performance from response bias and related top‐down factors was not available to use in this study. However, these factors are important to the mental representation of bodily signals and their impact on exercise behaviour is what we sought to capture in this study. There have been several recent studies that reinforce the evidence base for heartbeat counting performance as a valid measure of perceptual sensitivity to interoceptive signals (Schulz et al. 2021; Pohl et al. 2021; Ferentzi, Wilhelm, and Köteles 2022). Further research is required to understand the influence of multisystem and multifactorial interoceptive processing during exercise and its influence on the attention given to different sources of interoceptive feedback.

## 5 Conclusion

This study is the first to examine the effect of cardiac interoception during both externally prescribed constant‐load and self‐regulated exercises. The results show that heartbeat discrimination accuracy is implicated in self‐regulated exercise behaviours, resulting in differences in relative power outputs at *light* exercise intensities as well as differing pacing strategies at *hard‐to‐very hard* exercise intensities. However, tolerance to constant‐load exercise at 80% peak power output was not associated with a person's cardiac interoceptive characteristics. These findings suggest that cardiac interoception differentially influences exercise behaviour depending on the intensity and the nature of task being completed. Further research is required to understand whether individual differences across other interoceptive axes might influence perception of sensory signals and exercise behaviour.

## Conflicts of Interest

The authors declare no conflicts of interest.

## Supporting information

Supporting Information S1
