# Supplementary material for: Having the Heart to Exercise Control: Cardiac Interoception Influences Self‐Paced Exercise Regulation
Source: Eur J Sport Sci. 2025 Feb 15;25(3):e12263. doi: 10.1002/ejsc.12263 (PMC11829706; doi:10.1002/ejsc.12263)
Supplement: Supplementary file 1 — Supporting Information S1 [file EJSC-25-e12263-s001.docx]

# Having the heart to exercise control: Cardiac interoception influences self-paced exercise regulation.

**Supplementary Materials**





Supplementary Material, Figure 1. Time-to-task failure (s) during a constant-load task at 80% of peak power output for good (GOOD) and poor (POOR) heartbeat perceivers. There was no significant difference between groups defined by cardiac interoceptive accuracy.

Supplementary Material. Table 1. Group characteristics for good (GOOD) and poor (POOR) heartbeat perceivers related to measures of interoceptive sensitivity and physiological parameters.

|  |  | GOOD  (N = 9) | POOR  (N = 11) | Test statistic | *p-*value | Hedges’ *g* |
| --- | --- | --- | --- | --- | --- | --- |
| Demographic Characteristics | Age (yrs) | 22 (21-26) | 22 (21-22.5) | 61.5^U^ | .37 | .45 |
|  | Stature (cm) | 174.3 ± 9.7 | 170.2 ± 7.7 | 1.1 | .31 | .45 |
|  | Mass (kg) | 71.7 ± 9.7 | 66.2 ± 11.6 | 1.2 | .27 | .49 |
|  | Sex | 5M, 4F | 6M, 5F | - | - | - |
| Interoception: Heartbeat Tracking (SU) | Accuracy | 0.72 ± 0.21 | 0.67 ± 0.24 | 0.4 | .66 | .21 |
|  | Confidence | 5.3 ± 2.1 | 3.0 ± 1.9 | 2.6 | .02* | 1.11 |
|  | Awareness | 0.25 ± 0.30 | 0.40 ± 0.58 | 0.7 | .49 | .30 |
| Interoception: Heartbeat Discrimination (SU) | Accuracy | 0.72 ± 0.08 | 0.42 ± 0.10 | 7.4 | < .01* | 3.14 |
|  | Confidence | 5.9 ± 1.5 | 4.6 ± 1.8 | 1.8 | .10 | .74 |
|  | Awareness | 0.56 ± 0.21 | 0.40 ± 0.11 | 2.1^#^ | .06 | .95 |
| Peak | PO (W) | 308 ± 73 | 256 ± 40 | 2.0 | .06 | .87 |
|  | $\dot{V}$O_2_ (L·min^-1^) | 3.71 ± 0.99 | 3.18 ± 0.51 | 1.6 | .13 | .67 |
| VT_1_ | PO (W) | 190 ± 71 | 141 ± 27 | 2.0 | .06 | .91 |
|  | $\dot{V}$O_2_ (L·min^-1^) | 2.22 ± 0.71 | 1.83 ± 0.30 | 1.7 | .12 | .71 |
| VT_2_ | PO (W) | 249 ± 67 | 203 ± 35 | 2.0 | .06 | .85 |
|  | $\dot{V}$O_2_ (L·min^-1^) | 3.01 ± 0.79 | 2.55 ± 0.58 | 1.5 | .17 | .65 |
| Heart Rate  (beats·min^-1^) | Rest | 59 ± 17 | 69 ± 10 | 1.6 | .14 | .71 |
|  | Peak | 182 ± 13 | 184 ± 13 | 0.4 | .67 | .15 |

Abbreviations: SU = standardised units, PO = power output, W = watts, $\dot{V}$O_2_ = oxygen consumption, L·min^-1^ = litres per minute, beats·min^-1^ = beats per minute, * Significant difference between groups *p* < .05. *U* = Mann-Whitney statistic. # *t*-statistic corrected for violations to Levene’s Test for Equality of Variances.

Supplementary Material. Table 2. Group characteristics for good (GOOD) and poor (POOR) heartbeat perceivers related to measures of cardiac interoception and physiological variables.

|  |  | GOOD  (N = 7) | POOR  (N = 8) | *t*-statistic | *p-*value | Hedges’  *g* |
| --- | --- | --- | --- | --- | --- | --- |
| Demographic Characteristics | Age (yrs) | 23 ± 3 | 23 ± 2 | .313 | .76 | >.01 |
|  | Stature (cm) | 178 ± 7 | 168 ± 8 | 2.617 | .02^*^ | 1.27 |
|  | Mass (kg) | 74.7 ± 8.7 | 62.8 ± 10.6 | 2.370 | .03^*^ | 1.15 |
|  | Sex | 5M, 2F | 2M, 6F | - | - | - |
| Interoception: Heartbeat Tracking (SU) | Accuracy | 0.69 ± 0.23 | 0.52 ± 0.31 | 1.159 | .26 | .58 |
|  | Confidence | 5.4 ± 2.3 | 5.2 ± 2.7 | .320 | .75 | .08 |
|  | Awareness | 0.18 ± 0.29 | 0.11 ± 0.68 | .763 | .46 | .12 |
| Interoception:  Heartbeat Discrimination (SU) | Accuracy | 0.72 ± 0.09 | 0.28 ± 0.12 | 7.646 | < .01^*^ | 3.86 |
|  | Confidence | 5.8 ± 1.7 | 4.4 ± 2.3 | 1.494 | .16 | .63 |
|  | Awareness | 0.53 ± 0.22 | 0.41 ± 0.14 | 1.783 | .10 | .62 |
| VT_1_ | PO (W) | 205 ± 74 | 139 ± 32 | 2.342 | .04^*^ | 1.12 |
|  | $\dot{V}$O_2_ (L·min^-1^) | 2.33 ± 0.77 | 1.83 ± 0.25 | 1.777 | .10 | .85 |
| VT_2_ | PO (W) | 258 ± 75 | 186 ± 38 | 2.426 | .03^*^ | 1.17 |
|  | $\dot{V}$O_2_ (L·min^-1^) | 3.08 ± 0.99 | 2.41 ± 0.41 | 1.906 | .08 | .86 |
| Peak | PO (W) | 321 ± 79 | 242 ± 41 | 2.503 | .03^*^ | 1.21 |
|  | $\dot{V}$O_2_ (L·min^-1^) | 3.83 ± 1.11 | 3.03 ± 0.52 | 1.833 | .09 | .89 |
| Heart Rate  (b·min^-1^) | Rest | 62 ± 17 | 71 ± 18 | 1.382 | .19 | .48 |
|  | Peak | 181 ± 11 | 188 ± 5 | 1.600 | .16 | .79 |

Data presented as mean ± SD, SU = standardised units, VT_1_ = first ventilatory threshold, VT_2_ = second ventilatory threshold, W = watts, L∙min^-1^ = litres per minute, b∙min^-1^ = beats per minute, * *p* < .05.

Supplementary Material. Table 3. Summary of the main effects within the ANOVA for measures of power output in the RPE10 condition. ANOVAs are presented separately by analysis of the first 5minutes (minutes 1-5, 1-min averages) and the final 15-minutes (minutes 10-20, 5-min averages).

| minutes 1-5 | *F* | GROUP *p* | ηp2 | *F* | TIME *p* | ηp2 | *F* | GROUPxTIME *p* | ηp2 |
| --- | --- | --- | --- | --- | --- | --- | --- | --- | --- |
| PO_abs_ | 0.7 | .42 | .04 | 4.8 | .02* | .21 | 1.2 | .30 | .06 |
| %Trial_mean_ | 6.2 | .02* | .26 | 5.8 | .01* | .24 | 0.7 | .54 | .04 |
| %POVT1 | 4.5 | .047* | .20 | 4.0 | .03* | .18 | 1.1 | .36 | .06 |
| %PO_peak_ | 4.6 | .046* | .20 | 3.7 | .04* | .17 | 1.1 | .33 | .06 |

| minutes 10-20 | *F* | GROUP *p* | ηp2 | *F* | TIME *p* | ηp2 | *F* | GROUPxTIME *p* | ηp2 |
| --- | --- | --- | --- | --- | --- | --- | --- | --- | --- |
| PO_abs_ | 0.0 | .92 | .00 | 1.2 | .31 | .06 | 4.6 | .02* | .20 |
| %Trial_mean_ | 6.2 | .02* | .02 | 2.0 | .15 | .10 | 3.8 | .03* | .18 |
| %POVT1 | 2.3 | .14 | .12 | 1.6 | .21 | .08 | 3.7 | .03* | .17 |
| %PO_peak_ | 1.9 | .18 | .10 | 1.5 | .23 | .08 | 4.4 | .02* | .20 |

**p* < .05

Supplementary Material. Table 4. Summary of the main effects within the ANOVA for measures of power output in the RPE16 condition. ANOVAs are presented separately by analysis of the first 5minutes (minutes 1-5, 1-min averages) and the final 15-minutes (minutes 10-20, 5-min averages).

| minutes 1-5 | *F* | GROUP *p* | ηp2 | *F* | TIME *p* | ηp2 | *F* | GROGROUPxTIME *p* | ηp2 |
| --- | --- | --- | --- | --- | --- | --- | --- | --- | --- |
| PO_abs_ | 9.7 | .01* | .35 | 1.1 | .31 | .06 | 5.8 | .02* | .24 |
| %Trial_mean_ | 0.0 | .98 | .00 | 1.5 | .24 | .08 | 5.6 | .02* | .24 |
| %POVT2 | 0.0 | .92 | .00 | 1.8 | .20 | .09 | 5.9 | .02* | .26 |
| %PO_peak_ | 2.1 | .16 | .11 | 1.6 | .23 | .08 | 6.7 | .01* | .27 |
|  |  |  |  |  |  |  |  |  |  |
| minutes 10-20 | *F* | GROUP *p* | ηp2 | *F* | TIME *p* | ηp2 | *F* | OGROUPxTIME  *p* | ηp2 |
| PO_abs_ | 9.5 | .01* | .34 | 1.1 | .32 | .06 | 1.6 | .23 | .08 |
| %Trial_mean_ | 0.0 | .98 | .00 | 1.1 | .33 | .06 | 0.8 | .40 | .04 |
| %POVT2 | 0.0 | .90 | .00 | 0.0 | .89 | .00 | 1.1 | .36 | .06 |
| %PO_peak_ | 3.3 | .09 | .15 | 0.5 | .51 | .03 | 1.2 | .30 | .06 |

**p* < .05

Supplementary Material. Table 5. Summary of the main effects within the ANOVA for heart rate (*f*_c_), oxygen consumption (V̇ O_2)_, and respiratory exchange ratio (RER). ANOVAs are presented separately by analysis of the first 5-minutes (minutes 1-5, 1-min averages) and the final 15-minutes (minutes 10-20, 5-min averages).

| minutes 1-5 |  | GROUP  *F p* | | ηp2 | *F* | TIME *p* | ηp2 | GROUPxTIME  *F p* η_p_^2^ | | |
| --- | --- | --- | --- | --- | --- | --- | --- | --- | --- | --- |
| *f*c | RPE10 | 5.6 | .03* | .24 | 6.9 | .01* | .28 | 2.3 | .13 | .12 |
|  | RPE16 | 2.2 | .15 | .11 | 364.6 | <.01* | .95 | 2.0 | .16 | .10 |
|  |  |  |  |  |  |  |  |  |  |  |
| V̇O_2_ | RPE10 | 0.6 | .45 | .03 | 16.5 | <.01* | .48 | 0.4 | .59 | .02 |
|  | RPE16 | 1.8 | .20 | .09 | 213.4 | <.01* | .92 | 1.1 | .34 | .06 |
|  |  |  |  |  |  |  |  |  |  |  |
| RER | RPE10 | 16.7 | <.01* | .48 | 0.5 | <.01* | .24 | 1.1 | .35 | .06 |
|  | RPE16 | 1.4 | .25 | .07 | 186.6 | <.01* | .91 | 1.7 | .19 | .09 |
|  |  |  |  |  |  |  |  |  |  |  |

| minutes 10-20  *F* | | | | GROUP  *p* | | η_p_^2^ *F* | | TIME *p* | | ηp2 | | GROUPxTIME  *F p* η_p_^2^ | | | |
| --- | --- | --- | --- | --- | --- | --- | --- | --- | --- | --- | --- | --- | --- | --- | --- |
| *f*_c_ | RPE10 | 2.8 | .11 | .14 | | 0.4 | .70 | .02 | | 2.8 | | .11 | .14 |  |  |
|  | RPE16 | 0.1 | .81 | .00 | | 0.1 | .80 | .00 | | 0.7 | | .44 | .04 |  |  |
|  |  |  |  |  | |  |  |  | |  | |  |  |  |  |
| V̇O_2_ | RPE10 | 1.4 | .26 | .07 | | 0.6 | .50 | .03 | | 1.4 | | .26 | .07 |  |  |
|  | RPE16 | 0.1 | .76 | .01 | | 0.0 | .91 | .00 | | 0.3 | | .61 | .02 |  |  |
|  |  |  |  |  | |  |  |  | |  | |  |  |  |  |
| RER | RPE10 | 16.8 | <.01* | .48 | | 0.2 | .75 | .01 | | 16.8 | | <.01* | .48 |  |  |
|  | RPE16 | 1.1 | .30 | .06 | | 10.1 | <.01* | .36 | | 0.1 | | .92 | .00 |  |  |
|  |  |  |  |  | |  |  |  | |  | |  |  |  |  |

**p* < .05

Supplementary Material. Table 6. Summary of the main effects within the ANOVA for heart rate (*f*_c_), oxygen consumption (V̇ O_2)_, and respiratory exchange ratio (RER) and Rate of Perceived Exertion (RPE) for the Time to Exhaustion test for GOOD and POOR groups over the TIME of the test.

|  | GROUP | | | | TIME | | | | GROUPxTIME | | | |
| --- | --- | --- | --- | --- | --- | --- | --- | --- | --- | --- | --- | --- |
|  | *F* | df | *p* | ŋ_p_^2^ | *F* | df | *p* | ŋ_p_^2^ | *F* | df | *p* | ŋ_p_^2^ |
| *f*_c_ (%Peak) | 2.30 | 1, 13 | .153 | 0.15 | 195.57 | 1.684, 21.869 | < .001* | 0.94 | 2.54 | 1.684, 21.869 | .109 | 0.16 |
| VO_2_ (%Peak) | 1.81 | 1, 13 | .910 | <.01 | 113.67 | 1.232, 16.017 | < .001* | 0.90 | 1.29 | 1.232, 16.017 | .283 | 0.09 |
| RER (s.u.) | 4.29 | 1, 13 | .059 | 0.25 | 69.71 | 1.536, 19.972 | < .001* | 0.84 | 5.70 | 1.536, 19.972 | .016* | 0.28 |
| RPE (6-20) | 3.02 | 1, 13 | .106 | 0.19 | 106.48 | 1.696, 22.056 | < .001* | 0.89 | 0.64 | 1.696, 22.056 | .512 | 0.05 |

**p* < .05

Supplementary Material. Table 7. self-reported RPE data (median and range) for the Good and Poor groups at each of the time points it was recorded during the RPE10 and RPE16 trials in Experiment 1.

| Condition | Group |  | Time (min) |  |
| --- | --- | --- | --- | --- |
|  |  | 5 | 10 15 | 20 |
| RPE10 (a.u.) | GOOD | 10 (9 - 10) | 10 (10 - 10) 10(10-10) | 10(10-10) |
|  | POOR | 10 (10 - 11) | 10 (10 - 10.5) 10 (10 - 10) | 10 (10 - 10) |
|  |  |  |  |  |
| RPE16 (a.u.) | GOOD | 14 (13 – 15) | 16 (15 – 16) 16 (16 – 17) | 16 (16 – 16) |
|  | POOR | 15 (15 – 15.5) | 16 (16 – 16) 16 (16 – 16) | 16 (16 – 16) |
